# Association between Gastroesophageal Reflux Disease and Elastographic Parameters of Liver Steatosis and Fibrosis: Controlled Attenuation Parameter and Liver Stiffness Measurements

**DOI:** 10.1155/2021/6670065

**Published:** 2021-02-23

**Authors:** Ivana Mikolasevic, Goran Poropat, Tajana Filipec Kanizaj, Nadija Skenderevic, Marko Zelic, Marija Matasin, Luka Vranic, Andrea Kresovic, Goran Hauser

**Affiliations:** ^1^Department of Gastroenterology, Clinical Hospital Center Rijeka, Rijeka, Croatia; ^2^Department of Gastroenterology, University Hospital Merkur, Zagreb, Croatia; ^3^Faculty of Medicine, Rijeka, Croatia; ^4^Faculty of Medicine, Zagreb, Croatia; ^5^Faculty of Health Studies, Rijeka, Croatia; ^6^Department of Abdominal Surgery, Clinical Hospital Centre Rijeka, Croatia

## Abstract

**Aim:**

Our aim was to investigate the association among elastographic parameters of liver steatosis and fibrosis, controlled attenuation parameter (CAP) and liver stiffness measurement (LSM), with gastroesophageal reflux disease (GERD).

**Methods:**

In this prospective, cross-sectional study, we have evaluated 937 patients with one or more components of the metabolic syndrome who had an esophagogastroduodenoscopy (EGD) due to GERD symptoms. In all patients, a laboratory analysis, an abdominal ultrasound, and FibroScan measurements were done. GERD was defined by EGD.

**Results:**

The mean body mass index (BMI) of the study population was 30.95 ± 5.45 kg/m^2^. The prevalence of increased CAP was 82.6% (774/937). Patients with increased CAP were younger, were more obese, had higher prevalence of hypertension, diabetes, and dyslipidemia, and had higher values of aminotransferases. Similar results of higher prevalence in patients with elevated CAP were observed with GERD, hiatal hernia, and insufficient cardia (defined as deficient or absent closure of the gastric inlet in relation to the esophagus). Additionally, patients with elevated CAP had a higher prevalence of GERD grades B and C in comparison to those without elevated CAP. Consequently, patients who did not have elevated CAP had a higher prevalence of GERD grade A. Even though we have found an upward trend in the prevalence of GERD, hiatal hernia, and insufficient cardia, there was no significant difference between subjects with fibrosis (F) 1-2 and F3-4 stage of fibrosis or F1 and F2-4. In a binary logistic regression, a significant positive association with GERD was obtained for CAP. Furthermore, a significant positive association with hiatal hernia was obtained for BMI and CAP. Finally, a significant positive association with hiatal hernia was obtained with CAP in multivariate analysis.

**Conclusion:**

To the best of our knowledge, our study is the first to reveal a positive association between CAP as a surrogate marker of liver steatosis and GERD after adjustments for other clinical variables.

## 1. Introduction

According to data, about 25% of all cancers are in the gastrointestinal tract (GIT), making it the dominant cancer affected site [1]. As it is the case with most human tumors, esophageal carcinoma (EAC) is preceded by premalignant lesion or Barrett esophagus (BE). The main characteristic of BE is abnormal transformation of the squamous epithelium. Gastroesophageal reflux disease (GERD) is one of the most common GIT-related diseases worldwide and one of the most common indications for visiting gastroenterologists [1–7]. In the context of GERD, reflux of stomach contents into esophagus is responsible for the most common symptoms of this condition: heartburn, regurgitation, and dysphagia. A major concern of physicians who manage patients with GERD is the increased risk of EAC; thus, GERD is the most important risk factor for BE and EAC development [2–6]. The prevalence of GERD in general population is about 30% with an increasing overall, which is not surprising regarding the data that obesity (especially abdominal obesity) and the metabolic syndrome (MetS) are risk factors for GERD development [6–10].

Nonalcoholic fatty liver disease (NAFLD) is an increasingly growing cause of end-stage liver disease (i.e., liver cirrhosis and hepatocellular carcinoma (HCC)) and is the most common cause of chronic liver disease (CLD) today [11]. NAFLD is a clinical syndrome characterized by liver steatosis in individuals with no history of alcohol abuse, comprised of a spectrum of disorders. Histologically, there are few disorders in the context of NAFLD; for the first, there is simple steatosis, then necroinflammatory form called nonalcoholic steatohepatitis (NASH), then fibrosis and advanced fibrosis, and finally, cirrhosis. Normally, HCC is predisposed with the presence of cirrhosis, but in the context of NAFLD, HCC can evolve in non-cirrhotic NAFLD [11–13]. NAFLD is closely connected with the MetS and its individual components, diabetes mellitus type 2 (T2DM), arterial hypertension, obesity, and dyslipidemia [11–13]. The prevalence of NAFLD goes hand in hand with the prevalence of MetS and obesity due to its multisystemic effect; this combination is connected with the most serious health threat responsible for increasing number of chronic kidney diseases, cardiovascular, oncologic, and liver-related morbidity and mortality [12, 14]. In everyday clinical practice, the diagnosis of NAFLD represents a clinical challenge because most of NAFLD patients are asymptomatic. Although it is not the optimal method, liver biopsy (LB) is still the gold standard for the diagnosis and staging of NAFLD. Since around 25% of the population has NAFLD, noninvasive methods are being intensively investigated. The most investigated among elastographic methods is transient elastography (TE). With the help of controlled attenuation parameter (CAP) and liver stiffness measurement (LSM) obtained by TE, we can detect and quantify steatosis and fibrosis [15]. According to a recent study, CAP and LSM are good noninvasive methods for the assessment of steatosis and fibrosis in patients with NAFLD [16].

The relationship between NAFLD and GERD is controversial and published data are conflicting. According to some authors, there is no connection between these two conditions [5], while some others have found that GERD and its symptoms are more prevalent in NAFLD patients [6, 9, 10, 17]. According to our best knowledge, there are no published manuscripts that investigated the association between GERD and elastographic parameters of liver steatosis and fibrosis: CAP and LSM.

Therefore, our aim was to investigate the association among elastographic parameters of liver steatosis and fibrosis, CAP and LSM, with GERD.

## 2. Patients and Methods

### 2.1. Patients

In this prospective, cross-sectional study, we have evaluated 1050 patients with one or more components of the MetS who had an esophagogastroduodenoscopy (EGD) due to GERD symptoms (heartburn, regurgitation, and dysphagia) during the 24-month period between January 2018 and December 2019. In all patients, a laboratory analysis, an abdominal ultrasound (US), and FibroScan measurements were done. Patients who signed informed consent forms and were older than 18 years were part of this investigation. Patients with incomplete data, those who refused to undergo TE or US examination, those with significant alcohol consumption (>20 g per day for men and >10 g per day for women), other CLD (viral, metabolic, or autoimmune), celiac disease, and those with secondary causes of fatty liver such as drugs (amiodarone and tamoxifen) were excluded from the final analysis. Additionally, active malignancy, congestive heart failure and valvular heart disease, TE failure, and pregnancy were additional exclusion criteria. Because of these exclusion criteria, 937 patients were included in the final analysis. The Clinical Hospital Rijeka Ethics committee approved this research. Appropriate informed consent forms were signed by all patients. We conducted the research in accordance and agreement with the International Conference on Harmonization guidelines on Good Clinical Practice and with the Declaration of Helsinki.

### 2.2. Outcomes

The primary outcome of this study was to evaluate the association among elastographic parameters of liver steatosis and fibrosis, CAP and LSM, with the presence of GERD, hiatal hernia, insufficient cardia, and BE. Secondary outcomes were to investigate the association of GERD, hiatal hernia, BE, and insufficient cardia with laboratory, demographical data and elastographic parameters.

### 2.3. Clinical Assessment

In all analyzed patients, information on medical history and current drugs was recorded as well as demographic (age, sex, smoking, and alcohol consumption) and anthropometric (body mass index (BMI), waist circumference (WC), hip circumference (HC), and upper arm circumference (UAC)) data. Smoking was classified as nonsmoker, ex-smoker, and smoker. In all patients, information regarding the presence of one or more MetS components was analyzed. BMI was calculated as weight (kg)/height (m^2^). Hypertension was defined if the average blood pressure (after three repeated measures) was ≥140/90 mmHg, if there was positive medical history, or if the patient was taking anti-hypertensive drugs. Diabetes was defined as a fasting plasma glucose level ≥5.6 mmol/L or previously diagnosed T2DM or use of any hypoglycemic drugs. Dyslipidemia was defined as positive medical history, using of lipid-lowering drugs, or if the serum total cholesterol level was ≥5.2 mmol/L, serum triglyceride (TG) level ≥1.7 mmol/L, serum high-density lipoprotein (HDL) cholesterol level <1.0 mmol/L for male or <1.3 mmol/L for female, or serum low-density lipoprotein (LDL) cholesterol level ≥3.4 mmol/L.

An extensive laboratory evaluation was done in each patient in the morning hours after overnight fasting at the day of TE examination. Blood samples were collected from the patients to determine the full blood count and serum levels of liver enzymes (aspartate aminotransferase (AST), alanine aminotransferase (ALT), gamma-glutamyl transferase (GGT), and alkaline phosphatase (ALP)), fasting plasma glucose and fasting insulin, lipidogram (total cholesterol, LDL and HDL cholesterol, and triglycerides), renal tests (urea, creatinine), ferritin, serum uric acid, and C-reactive protein (CRP). Apart from a routine laboratory, each patient was screened for viral and other causes of CLD (metabolic and autoimmune). Well-trained nurses were responsible for measurements of anthropometry, blood pressure, and blood sampling.

### 2.4. Transient Elastography and Ultrasound Examination

Abdominal ultrasound examination was performed by an experienced specialist (gastroenterologist) with the help of Philips Affiniti (PC Best, Netherlands). As mentioned, in all patients TE examination after overnight fasting was done by using FibroScan® 502 Touch (Echosense, Paris, France), which was performed using M or XL probe by an experienced gastroenterologist. The examination was defined as valid if there were ≥10 valid measurements with interquartile range- (IQR-) to-median ratio of LSM ≤0.3. The diagnosis of liver steatosis was considered in patients with CAP ≥ 238 dB/m [18]. On the contrary, patients with LSM ≥7 kPa were defined to have a significant liver fibrosis (≥F2), while an advanced fibrosis (≥F3) was considered if LSM was ≥9.6 kPa using the M probe or ≥9.3 kPa using the XL probe. Finally, patients with LSM ≥11.5 kPa using the M probe or ≥11.0 kPa using XL probe were defined as having cirrhosis. These cutoff values were taken from the earlier data [19, 20]. TE was done within a month of EGD.

### 2.5. Esophagogastroduodenoscopy

All included patients had EGD which was done by an experienced gastroenterologist with the help of EVIS EXERA III Gastroscope (Olympus, Tokyo, Japan). The diagnosis of GERD was made only on basis of EGD finding. Patients without endoscopic changes, but with GERD symptoms, were not characterized “as having GERD.” The severity of GERD was defined according to the Los Angeles Classification. Hiatal hernia was defined if proximal dislocation of the gastroesophageal junction >2 cm above the diaphragmatic indentation [10, 21].

### 2.6. Statistical Analysis

Categorical variables are shown as percentages and continuous variables as means with standard deviation or medians with inter-quartile range. Difference between groups was tested using *χ*^2^-test for categorical variables and *t*-test or Mann–Whitney where appropriate for continuous variables. Binary logistic regression was performed in order to identify parameters independently associated with occurrence of GERD. All statistical analyses were performed using SPSS V.27.0 (SPSS Inc, Chicago, IL, USA). Statistical tests were two-tailed and significance was set at 0.05.

## 3. Results

### 3.1. Demographic and Laboratory Characteristics of Study Subjects Divided by Controlled Attenuation Parameter (CAP) for Liver Steatosis

Demographic and laboratory characteristics of all 937 study subjects and the characteristics of the subjects classified according to CAP categories are listed in Table 1. The median age of the whole group was 49 (46–66) years. Women were more represented (54% vs. 46%). The mean BMI of the study population was 30.95 ± 5.45 kg/m^2^, while the mean WC was 105.51 ± 14.56 cm. The prevalence of NAFLD based on TE-CAP was 82.6% (774/937). In Table 1, there are patient characteristics with and without increased CAP. Briefly, those with increased CAP were younger and had higher BMI, WC, HC, and UAC, higher prevalence of hypertension, T2DM, and dyslipidemia. Regarding the laboratory examinations, patients with elevated CAP had higher values of liver test (AST and ALT), ferritin, serum uric acid, and fasting insulin. Similar results of higher prevalence in patients with elevated CAP were observed with GERD, hiatal hernia, and insufficient cardia. Additionally, patients with elevated CAP had higher prevalence of GERD grades B and C in comparison to those without elevated CAP. Consequently, patients who did not have elevated CAP had higher prevalence of GERD grade A.

### 3.2. Prevalence of Outcomes among Subjects Divided by Stages of Liver Fibrosis (Ranges F1 to F4)

Even though we have found an upward trend in prevalence of GERD, hiatal hernia, and insufficient cardia, there was no significant difference between subjects with fibrosis (F)1-2 and F3-4 stage of fibrosis or F1 and F2-4 (defined by LSM). Prevalence according to the stage of liver fibrosis is shown in Table 2.

### 3.3. Association of GERD, Hiatal Hernia, Barrett's Esophagus, and Insufficient Cardia with Laboratory, Demographical Data and Elastographic Parameters: A Binary Logistic Regression

In a binary logistic regression, significant positive association with GERD was obtained for CAP. Results of this analysis are shown in Table 3. Furthermore, significant positive association with hiatal hernia was obtained for BMI, HDL cholesterol, and CAP (Table 4). Finally, significant positive association with hiatal hernia was obtained with CAP in multivariant analysis (Table 5). There was no association confirmed in multivariate analysis for Barrett's esophagus, due to the small number of patients with Barrett (Table 6).

## 4. Discussion

To the best of our knowledge, our study is the first to reveal a positive association between CAP as a surrogate marker of liver steatosis and GERD after adjustments for other clinical variables. Moreover, patients with elevated CAP had significantly higher prevalence of higher GERD grades (B and C). Similar result was published by other authors [3, 6, 17]. For example, in the study by Hung WC et al. [17], which was published a few years ago, there was a positive association between NAFLD and erosive esophagitis independent of obesity. In their study, NAFLD was diagnosed based on abdominal ultrasound [17]. However, ultrasound is a good method for the detection of moderate-severe fatty liver, but the sensitivity of ultrasound decreases with the decrement of fatty infiltration, so in the presence of a hepatic fat content of 10% to 19%, it had a sensitivity of only 55% shown in a study on 100 living liver donor candidates [22, 23]. In our study, patients with elevated CAP were more obese; however, in a binary logistic regression, only elevated CAP values were associated positively with GERD. Thus, it is possible that elevated CAP (i.e., NAFLD) have a greater impact on the risk of GERD than obesity. Similar observation was reported by another earlier mentioned study [17] and by a recent meta-analysis [3]. Also, Fujikawa et al. [10] showed that severer GERD symptoms in NAFLD compared to the controls were observed independently of degree of BMI. On the other hand, some authors did not confirm the connection among NAFLD and GERD [5]. However, further studies on this topic are needed.

Obesity is a well-known risk factor for GERD. We know from the earlier data that adipose tissue is metabolically active tissue that produces various inflammatory cytokines. Those cytokines relate to complications of GERD [24]. Other important factors that are involved in the pathogenesis of obesity and GERD are higher number of transient relaxations of the lower esophageal sphincter, the increased prevalence of esophageal motor disorders, and increased intra-abdominal pressure [24]. Our results showed that elevated CAP as a surrogate marker of liver steatosis (i.e., NAFLD) was associated with GERD. Our results raise the question of whether NAFLD can be involved in the pathogenesis of GERD. For over a century and a half, the important role of liver in the context of metabolism regulation has been recognized. However, fatty liver has for a long time been considered a trivial finding and just during the last 5–10 years the importance of NAFLD, not only for liver-related morbidity and mortality but as a condition that is connected to many extrahepatic diseases and cancers, has been recognized [25, 26]. NAFLD could be related to GERD via several mechanisms. Firstly, today we know from earlier data that in NAFLD patients there is an increased production of various proinflammatory cytokines, for example, interleukin-1 (IL-1), interleukin-6 (IL-6), TNF-alfa, TGF-beta, plasminogen activator inhibitor-1, increased reactive oxygen species, etc. These cytokines are produced by hepatocytes and non-parenchymal cells (Kupffer cells and hepatic stellate cells) [25–27]. It has been proposed that cytokines such as IL-1 and IL-6 could contribute to GERD development. Secondly, it is hypothesized that enhanced oxidative stress could lead to depletion of the adherent mucus layer and consequently damage esophageal mucosa. On the other hand, decreased antioxidant capacity is less able to prevent damage of esophageal mucosa and because of that the severity of GERD is increased [17, 28, 29]. And in the context of NAFLD there is increased systemic oxidative stress and a lower antioxidant capacity [17]. Thirdly, earlier data have shown that triglyceride could affect the lower esophageal sphincters. Hypertriglyceridemia is strongly associated with NAFLD and some authors have reported that this could be the shared underlying factor between GERD and NAFLD [30–33]. In our study, patients with elevated CAP had higher prevalence of dyslipidemia; however, we did not find serum triglyceride to be associated with GERD. But regarding the fact that patients were taking statins, we did not expect to find that association. Fourthly, autonomic nervous system dysfunction could represent additional link among NAFLD and GERB, because some data have reported that in NAFLD patients there is a higher prevalence of autonomic disturbance. On the other hand, it has been reported that autonomic dysfunction could be responsible for the abnormal gastric and esophageal motility and consequently it may predispose to development of GERD [3, 34, 35]. Finally, NAFLD is associated with obesity, especially with central obesity. We know that visceral fat is a metabolic active tissue responsible for releasing of proinflammatory cytokines. Also, regarding the fact that most of NAFLD patients are obese, there is direct mechanical effect on increasing gastric pressure which for the consequence has often lower esophageal sphincter relaxation with reflux of gastric acid [7, 17, 36]. Thus, enhanced oxidative stress and subchronic inflammatory state with release of inflammatory cytokines in the NAFLD patients, as well as strong correlation of NAFLD with central obesity, connect NAFLD with GERD development [17, 25, 26]. But further studies that will better investigate this association are needed.

Furthermore, the presence of insufficient cardia and hiatal hernia is associated with GERD [24]. In our study, obesity (defined by BMI) and CAP were independent predictors of presence of hiatal hernia. These results are in line with the connection of NAFLD and obesity. Recently, we have shown that CAP as a surrogate marker of NAFLD is correlated with MetS, obesity, and other MetS components [37, 38].

Finally, we had investigated the relationship of LSM as a surrogate marker of liver fibrosis with the presence insufficient cardia, hiatal hernia, GERD, and BE. Although there was no significant difference between subjects F1-2 and F3-4 stage of fibrosis or F1 and F2, we have found an upward trend in prevalence of GERD, hiatal hernia, and insufficient cardia according to the stage of liver fibrosis. We believe that with a larger number of patients this could reach a statistical significance. This data is in accordance with earlier observation that NAFLD is a multisystem disease and that degree of fibrosis is the strongest factor related to extrahepatic diseases that relate to NAFLD [14]. Further investigations on this topic are needed.

Nevertheless, our study has some limitations. Firstly, the cross-sectional design of the study precludes any causal inferences about the directionality of the connections that were found in our study. By the design of our study, we cannot exclude that apparent association among CAP (i.e., NAFLD) and GERD may not be causal but is a result of shared underlying risk factors (i.e., metabolic risk factors). Secondly, we did not use LB for NAFLD diagnosis. However, LB is an invasive procedure, and it would be non-ethical to perform LB in these patients. We used one of the best and widely available non-invasive methods that was reported as a good method for noninvasive assessment of liver steatosis and fibrosis [16]. According to our best knowledge, this is the first study in Croatia, in this part of Europe, which investigated the association among NAFLD and GERD. Thus, we have analyzed our single-center experience and our cohort should not be considered strictly representative of the general population. Furthermore, earlier data reported negative association among GERB and *Helicobacter pylori* infections [39]. In our cohort, we did not have information regarding this infection. There was no association confirmed in multivariate analysis for Barrett's esophagus, due to the small number of patients with BE; thus, further and larger studies are needed. However, our study was the first to date that investigated the association among elastographic parameters of liver steatosis and fibrosis (i.e., CAP and LSM) and GERD. It has the strength of a relatively large sample size and we use CAP and LSM obtained by TE that are one of the best validated non-invasive methods for the assessment of liver steatosis and fibrosis. Moreover, CAP and LSM measurements were assessed by using both FibroScan probes (M and XL). Finally, GERD was defined by “gold standard,” i.e., esophagogastroduodenoscopy.

In conclusion, our results demonstrate a significant association among CAP and GERD. Thus, in everyday clinical practice, we should pay more attention to NAFLD patients as they probably have an increased GERD risk. Further, longitudinal studies that will investigate this association and that will help us to understand underlying mechanisms between NAFLD and GERD are needed. Also, further studies could answer the question of whether by the use of noninvasive method (CAP and LSM) we could recognize those with GERD, especially those with severe forms that should undergo upper gastrointestinal endoscopy in order to prevent GERD complications, i.e., BE and esophageal cancer. This is important regarding the fact that, with the increase in incidence of obesity and MetS, the incidence of NAFLD is also increasing, and consequently, we can expect an increase in GERD incidence as well.

## Figures and Tables

**Table 1:** Demographic, laboratory, elastographic, and endoscopic characteristics of study subjects.

	All (*n* = 937)	Group 1 (*n* = 163), CAP < 238	Group 2 (*n* = 774), CAP ≥ 238	*p* value
Age, years (IQR)	49 (46–66)	53 (46–74)	47 (46–64)	0.007 ^*∗*^
Gender				
Male, *n* (%)	431 (46)	67 (41)	364 (47)	0.190
Female, *n* (%)	506 (54)	96 (59)	410 (53)	0.190
Smokers				
Nonsmokers, *n* (%)	654 (69.80)	113 (69.33)	541 (69.89)	0.962
Active, *n* (%)	190 (20.28)	28 (17.18)	162 (20.93)	0.329
Ex, *n* (%)	93 (9.92)	22 (13.49)	71 (9.17)	0.125
Body height (cm)	169.53 ± 10.15	168.15 ± 9.94	169.81 ± 10.18	0.057
Body weight (kg)	89.10 ± 17.92	78.55 ± 18.22	91.28 ± 17.05	<0.001 ^*∗*^
BMI (kg/m^2^)	30.95 ± 5.45	27.64 ± 5.16	31.62 ± 5.27	<0.001 ^*∗*^
Waist circumference (cm)	105.51 ± 14.56	96.94 ± 15.17	107.14 ± 13.88	<0.001 ^*∗*^
Hip circumference (cm)	110.24 ± 12.39	104.15 ± 13.51	111.4 ± 11.83	<0.001 ^*∗*^
Upper arm circumference (cm)	32.86 ± 6.6	30.28 ± 4.43	33.35 ± 6.85	<0.001 ^*∗*^
Haemoglobin (g/L)	136.79 ± 18.01	129.51 ± 19.45	138.38 ± 17.29	<0.001 ^*∗*^
Ferritin (ng/mL)	147.30 ± 152.24	124.27 ± 141.21	151.86 ± 154.04	0.035 ^*∗*^
Thrombocytes	227.26 ± 64.79	225.73 ± 80.60	227.60 ± 60.90	0.738
Serum glucose (mmol/L)	7.34 ± 8.15	6.43 ± 4.04	7.57 ± 8.87	0.109
HbA1c (%)	7.18 ± 13.47	5.88 ± 1.2	7.46 ± 14.83	0.175
Serum uric acid (mmol/L)	352.80 ± 107.07	316.34 ± 115.72	359.55 ± 104.11	<0.001 ^*∗*^
AST (U/L)	30.11 ± 26.42	24.49 ± 11.37	31.21 ± 28.33	0.003 ^*∗*^
ALT (U/L)	35.79 ± 26.98	26.70 ± 19.94	37.59 ± 27.82	<0.001 ^*∗*^
ALP (U/L)	78.03 ± 39.16	82.20 ± 35.66	77.20 ± 39.80	0.138
GGT (U/L)	53.77 ± 58.66	48.39 ± 61.98	54.82 ± 57.98	0.204
Total cholesterol (mmol/L)	6.04 ± 9.82	4.82 ± 1.07	6.31 ± 10.81	0.079
HDL (mmol/L)	1.98 ± 8.67	1.56 ± 0.4	2.07 ± 9.51	0.494
LDL (mmol/L)	3.58 ± 8.67	2.74 ± 0.98	3.75 ± 9.53	0.177
Triglyceride (mmol/L)	2.34 ± 8.04	1.36 ± 1.80	2.54 ± 8.80	0.089
Albumin (g/L)	44.28 ± 7.73	44.14 ± 3.99	44.31 ± 8.33	0.799
CRP (mg/L)	5.56 ± 15.52	6.61 ± 17.87	5.34 ± 14.98	0.342
Serum insulin (pmol/L)	20.32 ± 24.51	12.03 ± 8.02	22.02 ± 26.34	<0.001 ^*∗*^
Arterial hypertension, *n* (%)	629 (67.13)	93 (57.06)	536 (69.25)	0.004 ^*∗*^
Diabetes mellitus, *n* (%)	339 (36.18)	41 (25.15)	298 (38.50)	0.002 ^*∗*^
Hyperlipoproteinemia, *n* (%)	568 (60.62)	76 (46.63)	492 (63.57)	<0.001 ^*∗*^
CAP (dB/m)	297.76 ± 61.56	198.28 ± 29.78	318.7 ± 43.33	<0.001 ^*∗*^
LSM (kPa)	6.72 ± 4.08	5.13 ± 2.62	7.06 ± 4.25	<0.001 ^*∗*^
GERD, *n* (%)	293 (31.27)	30 (12.88)	263 (33.98)	<0.001 ^*∗*^
GERD grade A, *n* (%)	193 (20.59)	24 (80)	169 (64.25)	0.041 ^*∗*^
GERD grade B, *n* (%)	48 (5.12)	2 (6.66)	46 (17.49)	0.013 ^*∗*^
GERD grade C, *n* (%)	47 (5.01)	2 (6.66)	45 (17.11)	0.015 ^*∗*^
GERD grade D, *n* (%)	5 (0.51)	2 (6.66)	3 (1.14)	0.182
Barrett's esophagus, *n* (%)	14 (1.49)	1 (0.61)	13 (1.6)	0.504
Hiatal hernia, *n* (%)	402 (42.9)	40 (24.54)	362 (46.77)	<0.001 ^*∗*^
Insufficient cardia, *n* (%)	445 (47.49)	53 (32.52)	392 (50.65)	<0.001 ^*∗*^

AST: aspartate aminotransferase; ALT: alanine aminotransferase; GGT: gamma-glutamyl transferase; HDL: high-density lipoprotein; LDL: low-density lipoprotein; CRP: C-reactive protein; CAP: controlled attenuation parameter; LSM: liver stiffness measurement; GERD: gastroesophageal reflux disease. ^*∗*^*p* < 0.05.

**Table 2:** Prevalence of outcomes, gastroesophageal reflux disease, Barrett's esophagus, hiatal hernia, and insufficient cardia, among subjects divided by stages of liver fibrosis (ranges F1 to F4).

	F1 (*n* = 663)	F2 (*n* = 133)	F3 (*n* = 36)	F4 (*n* = 105)
GERD, *n* (%)	190 (28.66)	43 (32.33)	15 (41.67)	36 (34.29)
BE, *n* (%)	7 (1.06)	7 (1.50)	2 (5.56)	3 (2.86)
HH, *n* (%)	274 (41.33)	57 (42.86)	19 (52.78)	52 (49.52)
INSUF, *n* (%)	313 (47.21)	60 (45.11)	21 (58.33)	51 (48.57)
	F1-2 (*n* = 796)	F2-4 (*n* = 274)	F3-4 (*n* = 141)	
GERD, *n* (%)	233 (29.27)	94 (34.31)	51 (36.17)	
BE, *n* (%)	9 (1.13)	7 (2.55)	5 (3.55)	
HH, *n* (%)	331 (41.58)	128 (46.72)	71 (50.35)	
INSUF, *n* (%)	373 (46.86)	132 (48.18)	72 (51.06)	
	F1-2 vs. F3-4 *p* value	F1 vs. F2-4 *p* value		
GERD	0.123	0.102		
BE	0.071	0.102		
HH	0.065	0.149		
INSUF	0.407	0.843		

GERD: gastroesophageal reflux disease; BE: Barrett's esophagus; HH: hiatal hernia; INSUF: insufficient cardia.

**Table 3:** Association of GERD and laboratory, demographic data, and elastographic parameters.

	OR	95% CI	*p* value
BMI (kg/m^2^)	1.00	0.95–1.05	0.955
Age (years)	1.01	0.99–1.03	0.543
AST (U/L)	1.00	0.99–1.01	0.730
ALT (U/L)	1.01	0.99–1.02	0.358
GGT (U/L)	1.00	0.99–1.00	0.606
Total cholesterol (mmol/L)	1.01	0.97–1.05	0.572
HDL (mmol/L)	0.76	0.40–1.45	0.407
LDL (mmol/L)	0.89	0.69–1.15	0.374
Triglyceride (mmol/L)	0.98	0.87–1.10	0.744
Serum insulin, pmol/L	1.00	0.99–1.01	0.873
Arterial hypertension, *n* (%)	1.33	0.76–2.34	0.319
Diabetes mellitus, *n* (%)	1.09	0.63–1.88	0.765
Hyperlipoproteinemia, *n* (%)	0.89	0.53–1.49	0.661
LSM (kPa)	1.00	0.95–1.06	0.964
CAP (dB/m)	1.01	1.01–1.02	<0.001 ^*∗*^

BMI: body mass index; AST: aspartate aminotransferase; ALT: alanine aminotransferase; GGT: gamma-glutamyl transferase; HDL: high-density lipoprotein; LSM: liver stiffness measurement; LDL: low-density lipoprotein; CAP: controlled attenuation parameter. ^*∗*^*p* < 0.05.

**Table 4:** Association of hiatal hernia and laboratory, demographic data, and elastographic parameters.

	OR	95% CI	*p* value
BMI (kg/m^2)^	1.05	1.006–1.094	0.024 ^*∗*^
Age (years)	1.00	0.983–1.020	0.894
AST (U/L)	1.01	0.990–1.026	0.396
ALT (U/L)	1.00	0.985–1.011	0.734
GGT (U/L)	1.00	0.993–1.002	0.224
Total cholesterol (mmol/L)	1.08	0.889–1.302	0.450
HDL (mmol/L)	1.38	1.053–1.799	0.019 ^*∗*^
LDL (mmol/L)	0.76	0.566–1.023	0.070
Triglyceride (mmol/L)	0.91	0.785–1.058	0.222
Serum insulin (pmol/L)	1.00	0.990–1.007	0.699
Arterial hypertension, *n* (%)	1.14	0.689–1.895	0.605
Diabetes mellitus, *n* (%)	0.93	0.578–1.490	0.757
Hyperlipoproteinemia, *n* (%)	1.04	0.656–1.633	0.884
CAP (dB/m)	1.01	1.003–1.010	0.001^∗^

BMI: body mass index; AST: aspartate aminotransferase; ALT: alanine aminotransferase; GGT: gamma-glutamyl transferase; HDL: high-density lipoprotein; LDL: low-density lipoprotein; CAP: controlled attenuation parameter. ^*∗*^*p* < 0.05.

**Table 5:** Association of insufficient cardia and laboratory, demographic data, and elastographic parameters.

	OR	95% CI	*p* value
BMI (kg/m^2^)	1.04	0.999–1.086	0.057
Age (years)	1.01	0.987–1.023	0.609
AST (U/L)	1.00	0.990–1.018	0.589
ALT (U/L)	1.00	0.986–1.009	0.672
GGT (U/L)	1.00	0.992–1000	0.068
Total cholesterol (mmol/L)	1.13	0.745–1.725	0.558
HDL (mmol/L)	1.17	0.901–1.519	0.238
LDL (mmol/L)	0.84	0.532–1.340	0.473
Triglyceride (mmol/L)	0.92	0.810–1.053	0.238
Serum insulin (pmol/L)	1.00	0.987–1.006	0.466
Arterial hypertension, *n* (%)	1.05	0.636–1.727	0.853
Diabetes mellitus, *n* (%)	0.89	0.555–1.418	0.617
Hyperlipoproteinemia, *n* (%)	1.01	0.640–1.584	0.977
CAP (dB/m)	1.01	1.003–1.011	0.001 ^*∗*^

BMI: body mass index; AST: aspartate aminotransferase; ALT: alanine aminotransferase; GGT: gamma-glutamyl transferase; HDL: high-density lipoprotein; LDL: low-density lipoprotein; CAP: controlled attenuation parameter. ^*∗*^*p* < 0.05.

**Table 6:** Association of Barrett's esophagus and laboratory, demographic data, and elastographic parameters.

	OR	95% CI	*p* value
BMI (kg/m^2^)	0.66	0.385–1.138	0.135
Age (years)	0.86	0.672–1.109	0.248
AST (U/L)	0.98	0.742–1.281	0.858
ALT (U/L)	1.02	0.877–1.191	0.783
GGT (U/L)	1.01	0.980–1.036	0.613
Total cholesterol (mmol/L)	0.97	0.716–1.323	0.861
HDL (mmol/L)	0.41	0.037–4.675	0.467
LDL (mmol/L)	4.69	0.591–37.216	0.144
Triglyceride (mmol/L)	0.56	0.081–3.859	0.556
Serum insulin (pmol/L)	1.03	0.951–1.111	0.486
Arterial hypertension, *n* (%)	1.00	—	0.994
Diabetes mellitus, *n* (%)	0.12	0.001–22.593	0.431
Hyperlipoproteinemia, *n* (%)	1.54	0.041–57.392	0.816
CAP (dB/m)	1.10	0.969–1.238	0.147

BMI: body mass index; AST: aspartate aminotransferase; ALT: alanine aminotransferase; GGT: gamma-glutamyl transferase; HDL: high-density lipoprotein; LDL: low-density lipoprotein; CAP: controlled attenuation parameter.

## Data Availability

Access to data is restricted due to ethical restrictions.

## References

[1] Mikolašević I., Bokun T., Filipec Kanižaj T. (2018). Gastroesophageal reflux disease, Barrett esophagus, and esophageal adenocarcinoma-where do we stand?. *Croatian Medical Journal*.

[2] Shaheen N., Ransohoff D. F. (2002). Gastroesophageal reflux, Barrett esophagus, and esophageal cancer. *JAMA*.

[3] Wijarnpreecha K., Panjawatanan P., Thongprayoon C., Jaruvongvanich V., Ungprasert P. (2017). Association between gastroesophageal reflux disease and nonalcoholic fatty liver disease: a meta-analysis. *Saudi Journal of Gastroenterology*.

[4] Xue J., Xin H., Ren N. (2019). Nonalcoholic fatty liver disease increases the risk of gastroesophageal reflux disease: a systematic review and meta‐analysis. *European Journal of Clinical Investigation*.

[5] Min Y. W., Kim Y., Gwak G.-Y. (2018). Non-alcoholic fatty liver disease and the development of reflux esophagitis: a cohort study. *Journal of Gastroenterology and Hepatology*.

[6] Catanzaro R., Calabrese F., Occhipinti S. (2014). Nonalcoholic fatty liver disease increases risk for gastroesophageal reflux symptoms. *Digestive Diseases and Sciences*.

[7] Chung S. J., Kim D., Park M. J. (2008). Metabolic syndrome and visceral obesity as risk factors for reflux oesophagitis: a cross-sectional case-control study of 7078 Koreans undergoing health check-ups. *Gut*.

[8] Niigaki M., Adachi K., Hirakawa K., Furuta K., Kinoshita Y. (2013). Association between metabolic syndrome and prevalence of gastroesophageal reflux disease in a health screening facility in Japan. *Journal of Gastroenterology*.

[9] Fujiwara M., Eguchi Y., Fukumori N. (2015). The symptoms of gastroesophageal reflux disease correlate with high body mass index, the aspartate aminotransferase/alanine aminotransferase ratio and insulin resistance in Japanese patients with non-alcoholic fatty liver disease. *Internal Medicine*.

[10] Fujikawa Y., Tominaga K., Fujii H. (2012). High prevalence of gastroesophageal reflux symptoms in patients with non-alcoholic fatty liver disease associated with serum levels of triglyceride and cholesterol but not simple visceral obesity. *Digestion*.

[11] Mikolasevic I., Filipec-Kanizaj T., Mijic M. (2018). Nonalcoholic fatty liver disease and liver transplantation - where do we stand?. *World Journal of Gastroenterology*.

[12] Grgurevic I., Podrug K., Mikolasevic I., Kukla M., Madir A., Tsochatzis E. A. (2020). Natural history of nonalcoholic fatty liver disease: implications for clinical practice and an individualized approach. *Canadian Journal of Gastroenterology and Hepatology*.

[13] Milić S., Lulić D., Štimac D. (2014). Non-alcoholic fatty liver disease and obesity: biochemical, metabolic and clinical presentations. *World Journal of Gastroenterology*.

[14] Mikolasevic I., Milic S., Turk Wensveen T. (2016). Nonalcoholic fatty liver disease-a multisystem disease?. *World Journal of Gastroenterology*.

[15] Mikolasevic I., Orlic L., Franjic N., Hauser G., Stimac D., Milic S. (2016). Transient elastography (FibroScan) with controlled attenuation parameter in the assessment of liver steatosis and fibrosis in patients with nonalcoholic fatty liver disease-where do we stand?. *World Journal of Gastroenterology*.

[16] Eddowes P. J., Sasso M., Allison M. (2019). Accuracy of FibroScan controlled attenuation parameter and liver stiffness measurement in assessing steatosis and fibrosis in patients with nonalcoholic fatty liver disease. *Gastroenterology*.

[17] Hung W.-C., Wu J.-S., Yang Y.-C., Sun Z.-J., Lu F.-H., Chang C.-J. (2014). Nonalcoholic fatty liver disease vs. obesity on the risk of erosive oesophagitis. *European Journal of Clinical Investigation*.

[18] Sasso M., Beaugrand M., de Ledinghen V. (2010). Controlled attenuation parameter (CAP): a novel VCTE guided ultrasonic attenuation measurement for the evaluation of hepatic steatosis: preliminary study and validation in a cohort of patients with chronic liver disease from various causes. *Ultrasound in Medicine & Biology*.

[19] Wong V. W., Vergniol J., Wong G. L.-H. (2010). Diagnosis of fibrosis and cirrhosis using liver stiffness measurement in nonalcoholic fatty liver disease. *Hepatology*.

[20] Wong V. W., Vergniol J., Wong G. L.-H. (2012). Liver stiffness measurement using XL probe in patients with nonalcoholic fatty liver disease. *American Journal of Gastroenterology*.

[21] Nam S. Y., Choi I. J., Ryu K. H., Park B. J., Kim H. B., Nam B. H. (2010). Abdominal visceral adipose tissue volume is associated with increased risk of erosive esophagitis in men and women. *Gastroenterology*.

[22] Hernaez R., Lazo M., Bonekamp S. (2011). Diagnostic accuracy and reliability of ultrasonography for the detection of fatty liver: a meta-analysis. *Hepatology*.

[23] Ryan C. K., Johnson L. A., Germin B. I., Marcos A. (2002). One hundred consecutive hepatic biopsies in the workup of living donors for right lobe liver transplantation. *Liver Transplantation*.

[24] Chang P., Friedenberg F. (2014). Obesity and GERD. *Gastroenterology Clinics of North America*.

[25] Mikolasevic I., Racki S., Lukenda V., Pavletic-Persic M., Milic S., Orlic L. (2014). Non-alcoholic fatty liver disease; a part of the metabolic syndrome in the renal transplant recipient and possible cause of an allograft dysfunction. *Medical Hypotheses*.

[26] Mikolasevic I., Racki S., Zaputovic L., Lukenda V., Milic S., Orlic L. (2014). Nonalcoholic fatty liver disease (NAFLD): a new risk factor for adverse cardiovascular events in dialysis patients. *Medical Hypotheses*.

[27] Targher G., Bertolini L., Rodella S. (2008). NASH predicts plasma inflammatory biomarkers independently of visceral fat in men. *Obesity*.

[28] Cheng H. H., Chang C.-S., Wang H.-J., Wang W.-C. (2010). Interleukin-1*β* and -10 polymorphisms influence erosive reflux esophagitis and gastritis in Taiwanese patients. *Journal of Gastroenterology and Hepatology*.

[29] Rieder F., Biancani P., Harnett K., Yerian L., Falk G. W. (2010). Inflammatory mediators in gastroesophageal reflux disease: impact on esophageal motility, fibrosis, and carcinogenesis. *American Journal of Physiology-Gastrointestinal and Liver Physiology*.

[30] Wu P., Ma L., Dai G. X. (2011). The association of metabolic syndrome with reflux esophagitis: a case-control study. *Neurogastroenterology & Motility*.

[31] Ledeboer M., Masclee A. A. M., Biemond I., Lamers C. B. H. W. (1998). Effect of medium- and long-chain triglycerides on lower esophageal sphincter pressure: role of CCK. *American Journal of Physiology-Gastrointestinal and Liver Physiology*.

[32] Shapiro M., Green C., Bautista J. M. (2007). Assessment of dietary nutrients that influence perception of intra-oesophageal acid reflux events in patients with gastro-oesophageal reflux disease. *Alimentary Pharmacology & Therapeutics*.

[33] Liu Y. C., Hung C. S., Wu Y. W. (2013). Influence of non-alcoholic fatty liver disease on autonomic changes evaluated by the time domain, frequency domain, and symbolic dynamics of heart rate variability. *PLoS One*.

[34] Chen C.-L., Orr W. C. (2004). Autonomic responses to heartburn induced by esophageal acid infusion. *Journal of Gastroenterology and Hepatology*.

[35] Devendran N., Chauhan N., Armstrong D., Upton A. R. M., Kamath M. V. (2014). GERD and obesity: is the autonomic nervous system the missing link?. *Critical Reviews in Biomedical Engineering*.

[36] Wu J. C. Y., Mui L. M., Cheung C. M. Y., Chan Y., Sung J. J. Y. (2007). Obesity is associated with increased transient lower esophageal sphincter relaxation. *Gastroenterology*.

[37] Mikolasevic I., Lukenda Zanko V., Jakopcic I. (2020). Prospective evaluation of non-alcoholic fatty liver disease by elastographic methods of liver steatosis and fibrosis; controlled attenuation parameter and liver stiffness measurements. *Journal of Diabetes and its Complications*.

[38] Mikolasevic I., Milic S., Orlic L., Stimac D., Franjic N., Targher G. (2016). Factors associated with significant liver steatosis and fibrosis as assessed by transient elastography in patients with one or more components of the metabolic syndrome. *Journal of Diabetes and its Complications*.

[39] Koike T., Ohara S., Sekine H. (2001). Helicobacter pylori infection prevents erosive reflux oesophagitis by decreasing gastric acid secretion. *Gut*.

